# Drug‐phospholipid conjugate nano‐assembly for drug delivery

**DOI:** 10.1002/SMMD.20240053

**Published:** 2024-12-22

**Authors:** Ding Zhao, Yixiang Zhang, Fan Wang, Rames Kaewmanee, Wenguo Cui, Tianqi Wu, Yawei Du

**Affiliations:** ^1^ Department of Orthopaedics Shanghai Key Laboratory for Prevention and Treatment of Bone and Joint Diseases Shanghai Institute of Traumatology and Orthopaedics Ruijin Hospital Shanghai Jiao Tong University School of Medicine Shanghai China; ^2^ Department of Plastic & Reconstructive Surgery Shanghai Ninth People's Hospital Shanghai Jiao Tong University School of Medicine Shanghai China; ^3^ Department of Materials Science Faculty of Science Chulalongkorn University Bangkok Thailand; ^4^ Department of Radiation Oncology Huashan Hospital Fudan University Shanghai China

**Keywords:** assembly, drug delivery, drug‐phospholipid conjugate, liposome, prodrug

## Abstract

Phospholipid‐based liposomes are among the most successful nanodrug delivery systems in clinical use. However, these conventional liposomes present significant challenges including low drug‐loading capacity and issues with drug leakage. Drug‐phospholipid conjugates (DPCs) and their assemblies offer a promising strategy for addressing these limitations. In this review, we summarize recent advances in the design, synthesis, and application of DPCs for drug delivery. We begin by discussing the chemical backbone structures and various design strategies such as phosphate head embedding and mono‐/bis‐embedding in the sn‐1/sn‐2 positions. Furthermore, we highlight stimulus‐responsive designs of DPCs and their applications in treating diseases such as cancer, inflammation, and malaria. Lastly, we explore future directions for DPCs development and their potential applications in drug delivery.

## Graphical abstract

Drug‐phospholipid conjugates (DPCs) possess the prodrug characteristics and self‐assembly abilities, making them a potential alternative to traditional phospholipids in the preparation of nanomedicines. The nano‐assemblies formed by DPCs exhibit decent advantages such as high drug‐loading capacity and strong stability, showing great translational potential in areas such as anti‐tumor, anti‐inflammation, anti‐malaria, anti‐epileptics and vaccines.

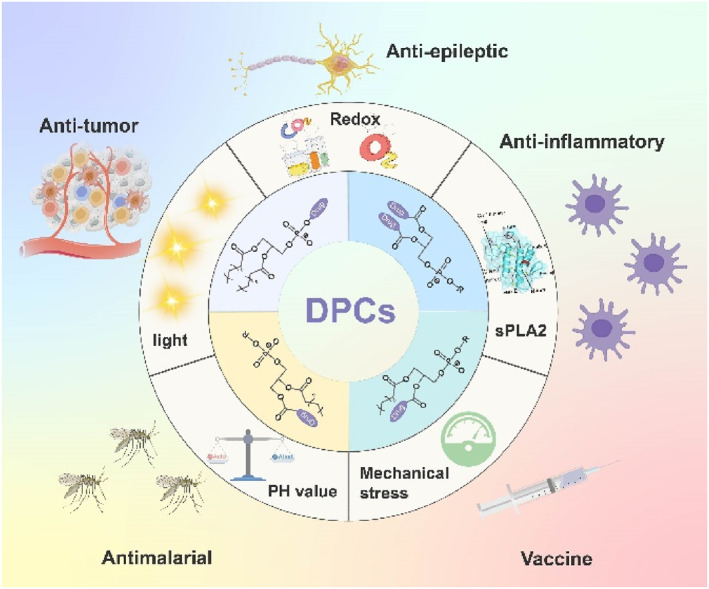

**Key points**

- Background and chemical structural feature of drug‐phospholipid conjugates (DPCs) are introduced.
- Stimulus‐responsive designs of DPCs and their applications in treating diseases are highlighted.
- Future directions for DPCs development and their potential applications in drug delivery are prospected.

## 1 INTRODUCTION

Typical phospholipids are a type of amphiphilic compounds with a hydrophilic phosphate head and two hydrophobic tails, which are the basic components of lipid‐bilayer membranes.^1^ In the 1960s, phospholipids were first discovered to be formulated into special nano‐vesicae with typical lipid‐bilayer structures for drug delivery, which were named “liposomes”.^2^ Liposomes based on phospholipids are absolutely the most successful nano drug delivery systems for clinical application.^3^ Traditional phospholipids are merely inert components in pharmaceutical formulations, and hydrophobic and hydrophilic drugs should be encapsulated in the lipid‐bilayer and aqueous inner core of the phospholipid‐assembled liposomes, respectively.^3^, ^4^, ^5^ However, the drawbacks of these traditional liposomes are obvious as well including low drug‐loading and leakage issues.^6^ Herein, the emerging drug‐phospholipid conjugates (DPCs) provide a unique approach to settle these problems.^7^

As the name implies, DPCs are conjugates of drug molecules and phospholipid structures. Similar strategies are widely reported, such as drug‐antibody,^8^ drug‐polymer,^9^ drug‐peptide^10^ or even drug‐drug conjugates,^11^ of which most can be classified as prodrugs. These prodrug conjugates would be inactive before metabolism or conversion, and the efficacy of drug active molecules could be primarily maintained. Different from these mentioned prodrug conjugates, DPCs are a kind of small molecules designed based on traditional phospholipids, which not only gives DPCs well‐defined molecular structures for potential clinical application but also ensures them excellent assembly capabilities due to structural similarities. Most studies indicate that DPCs can directly replace traditional phospholipids for the assembly of various nanostructures, including liposomes,^12^ micelles,^13^ solid lipid nanoparticles (SLNs)^14^, ^15^ and lipid nanoparticles (LNPs).^12^, ^16^ Although many amphiphilic compounds, such as polymers, have also been reported to possess similar assembly capabilities, the biomimicry of natural membrane structures assembled by phospholipid analogs like DPCs is unparalleled by other compounds.^13^ Firstly, the assemblies of DPCs derived from most hydrophobic drugs would maintain their polar heads facing outward, preserving the surface structural characteristics of traditional liposomes without altering their biocompatibility. Secondly, this similarity also allows us to prepare DPCs liposome‐like assemblies without the need for extensive adjustments to traditional liposome preparation processes.^12^ Undoubtedly, these direct assemblies by DPCs demonstrate dramatical properties compared to traditional liposomal agents, including extremely high drug‐loading, minimal leakage and reduced excipients.^17^ Therefore, DPCs are a kind of biomimetic phospholipid prodrug, particularly suitable for the construction of nano‐assemblies.

The application of DPCs and their nano‐assemblies is inseparable from the exploration of their stimuli‐triggered release mechanisms, which directly affects the design of DPCs.^7^ Among DPCs, the drug‐lysophospholipid conjugates with sn‐2 position replacement are the most widely studied. This is mainly because of the overexpressed secretory phospholipase A2 enzyme (sPLA2) in many lesions, such as tumor and inflammation, which specifically hydrolyzes the ester bond at the sn‐2 position of phospholipids.^18^, ^19^ Moreover, lysophospholipids are readily available, and their conjugation with pharmaceutical molecules is also relatively straightforward.^20^ The current design of DPCs has gone beyond the previous routine of sPLA2‐sensitive lysophospholipid conjugates. With the development of synthetic technology, the chemical reaction efficiency is no longer a problem for complex molecular designs. Different active structures could be conjugated or embedded onto the phospholipid skeleton. Owing to the diversity of conjugated pharmaceutical molecules, DPCs can play a variety of roles in the drug delivery process. Essentially, DPCs are prodrugs with assembly ability which could be used directly for monotherapy. From the other perspective, DPCs are also the alternatives of traditional phospholipids, and the assembled liposomes or liposome‐like vesicae could be regarded as medicative drug carriers for other combined compounds.^21^ In fact, the specific function of conjugated drug molecules would greatly enrich the usage scenarios of DPCs in drug delivery systems. DPCs could be applied as sensitive components for stimuli‐triggered drug release. For example, by conjugation of ferrocene onto the phosphate heads, DPCs would provide a redox sensitivity of the assembled liposomes as the desalt process under redox environment changes the hydrophilicity and then makes the assembly unstable.^22^ Besides, embedding photosensitizer porphyrin to replace the hydrophobic tail would not only provide liposomes photosensitiveness but also improve their affinity to histidine‐tagged proteins after the cobalt complexing.^23^

**FIGURE 1:**
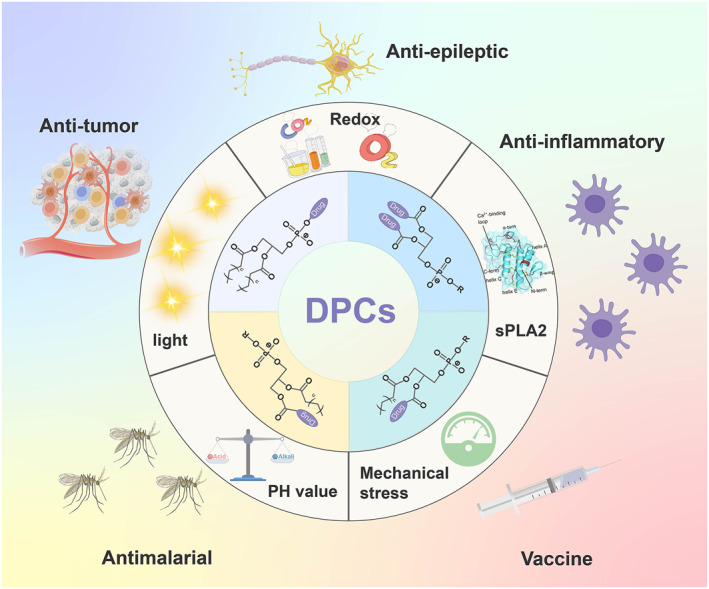
Illustration of the designs and applications of different DPCs.

In recent years, DPCs have attracted increasing attention in the drug delivery fields. These functional phospholipid mimics not only act as effective prodrugs but also can partially or completely replace traditional phospholipids in the preparation of novel liposomes or liposome analogs with high specific environmental sensitivity, high drug loading and high stability. In addition, more potential applications continue to be reported due to the diversity of conjugated active molecules. In this review, we focused on the chemical structure design of DPCs, including basic classification and stimuli‐triggered methods (Figure 1). Different applications of DPCs were also discussed for a clear presentation of the recent research status. Finally, we summarized the study of DPCs and DPC‐based liposomes and made a prospect for the future studies in this area.

## 2 CHEMICAL STRUCTURE OF DPCS

### 2.1 Glycerophospholipid (with glycerol backbone) based DPCs

**FIGURE 2:**
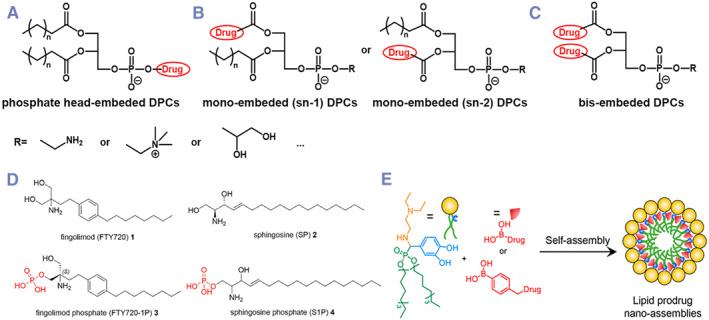
Different chemical structures of DPCs: (A) DPCs with phosphate head embedded drug molecules; (B) DPCs with mono‐embedded (sn‐1 or sn‐2) drug molecules and (C) DPCs with bis‐embedded (sn‐1 and sn‐2) drug molecules; (D, E) two examples of non‐glycerol based DPCs. Modified and reprinted under terms of the CC‐BY license.^25^ Copyright 2024, The Authors, published by Elsevier. Modified and reprinted with permission.^26^ Copyright 2023, American Chemical Society.

Generally, DPCs share a similar chemical structure with traditional phospholipids with a glycerol backbone (glycerophospholipids). According to the source, traditional glycerophospholipids can be divided into natural glycerophospholipids (such as lecithin) and synthetic glycerophospholipids. Typically, synthetic glycerophospholipids have the precise chemical structures, which could be further classified according to the polar phosphate head and hydrophobic tails. Most DPCs are constructed based on these synthetic glycerophospholipids.^24^ Until now, the reported glycerophospholipid based DPCs can be roughly divided into 3 types (Figure 2A–C): (1) with phosphate head embedded drug molecules, (2) with mono‐embedded (sn‐1 or sn‐2) drug molecules, and (3) with bis‐embedded (sn‐1 and sn‐2) drug molecules.

### 2.2 Non‐glycerol based DPCs

However, from a broad perspective, phospholipids are not limited to the glycerol skeleton. Even some phospholipids themselves do not have a glycerol backbone, such as sphingomyelin and its derivatives.^27^ In theory, DPCs could also be constructed based on such non‐glycerol phospholipids.

Bajaj et al. reported a Tamoxifen‐phospholipid conjugate (LCA‐Tam‐PC) with lithocholic acid backbone rather than classical glycerol backbone.^28^ This unique framework structure endows LCA‐Tam‐PC with better oral bioavailability, lower hepatotoxicity, and higher antitumor activity. In addition, the phospholipid structure could conjugate directly to active molecules. For example, Pertusati et al. reported a Fingolimod phosphoramidate prodrug (FTY720‐1P) for the treatment of multiple sclerosis. Similar to other classical DPCs, FTY720‐1P could also be assembled into lipid bilayers (Figure 2D).^25^ Additionally, there is another class of DPCs with phosphate modifications that still retain the traditional phospholipid‐like structure but lack a glycerol backbone. For example, Ding et al. reported a modular DPC structure based on the efficient covalent conjugation of boronic esters. After being modified with boronic acid, drugs can efficiently conjugate to the phospholipid‐like core structure (Figure 2E).^26^ Generally, this type of DPCs is less reported and lacks regularity.

## 3 DESIGN OF GLYCEROPHOSPHOLIPID BASED DPCS

### 3.1 Phosphate head embedded DPCs

**FIGURE 3:**
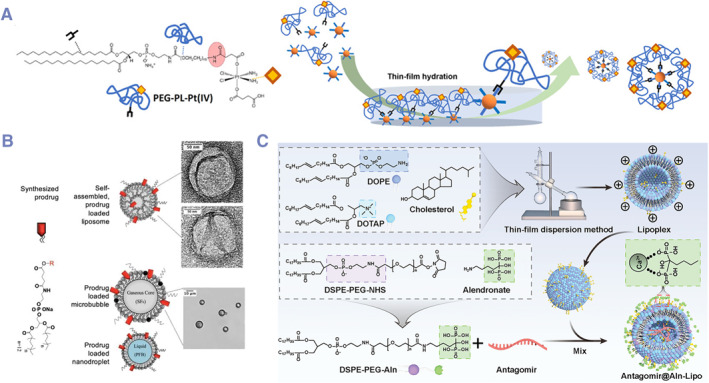
Phosphate head embedded DPCs: (A) the water‐soluble cisplatin can be conjugated directly to phospholipids. Modified and reprinted under terms of the CC‐BY license.^30^ Copyright 2023, The Authors, published by John Wiley and Sons; (B) liposomes based on hydrophobic drug‐embedded DPCs (phosphate head) could be destroyed easily under ultrasound, which could be used for targeted drug release. Modified and reprinted under terms of the CC‐BY license.^31^ Copyright 2020, The Authors, published by Ivyspring international publisher; (C) bisphosphonates are conjugated to a phospholipid structure via a PEG linker, forming this type of specialized PDCs, which can target mineralized bone surfaces. Modified and reprinted with permission.^32^ Copyright 2023, John Wiley and Sons.

Because the hydrophilic phosphate heads of most phospholipids have reactive amino and hydroxyl functional groups, it is relatively easy to conjugate drug molecules, and the amphiphilic structures of phospholipids will not be affected. Phosphate head embedded technology is relatively mature, and this related work has been reported widely. To avoid the influence on the assembly ability, hydrophilic drugs are mainly selected as active molecules in this type of DPCs, and hydrophobic drugs will affect the assembly performance of conjugates to some extent.^29^ For example, the water‐soluble anti‐HIV drug Zidovudine (AZT), the anti‐tumor drug Gemcitabine and cisplatin can be conjugated directly to phospholipids via ether bonds (Figure 3A).^30^, ^33^ By contrast, hydrophobic drugs for phosphate head embedded DPCs are rare, and most of them are designed for assembling sensitive liposomes due to their unstable properties. For example, Tartis et al. showed that liposomes based on hydrophobic drug‐embedded DPCs (phosphate head) could be destroyed easily under ultrasound, which could be used for targeted drug release (Figure 3B).^31^

Another kind of work uses hydrophilic chains as connecting arms (such as polyethylene glycol chain segment) to conjugate drugs to ensure the assembly performance of phospholipids. Although conjugation with connecting arms still can be classified to this type of DPC, the conjugated drugs are arranged outwardly with the outside of phospholipids, and they are inevitably exposed, and the drug‐loading would be limited. This conjugate was only appropriate for the conjugate of affinity ligands such as antibodies and peptides. Some molecules possess both therapeutic and targeting properties, making them particularly suitable for constructing PDCs using this strategy. For example, bisphosphonates are conjugated to a phospholipid structure via a PEG linker, forming this type of specialized PDCs, which not only have the ability to target mineralized bone surfaces but also prevent osteoclast‐mediated bone resorption (Figure 3C).^32^, ^34^ Generally, this strategy not only requires the conjugated drug to be relatively hydrophilic and stable but also to possess certain targeting capabilities.

### 3.2 Mono‐embedded (sn‐1 or sn‐2) DPCs

**FIGURE 4:**
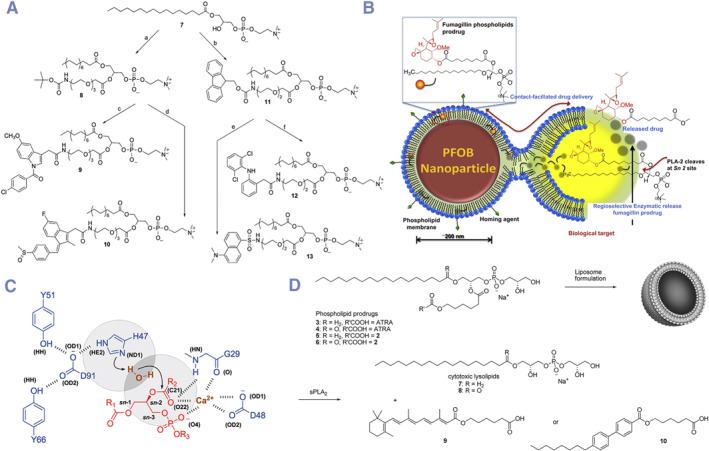
sPLA2 sensitive mono‐embedded (sn‐2) DPCs: (A) synthesis of non‐steroidal anti‐inflammatory drugs (NSAIDs) based sn‐2 mono‐embedded DPCs as sPLA2‐sensitive prodrugs. Modified and reprinted with permission.^37^ Copyright 2010, Elsevier; (B) membrane fusion and sPLA2 hydrolysis of fumagillin based sn‐2 mono‐embedded DPC. Modified and reprinted with permission.^38^ Copyright 2012, Elsevier; (C) the mechanism of sPLA2 hydrolysis of mono‐embedded (sn‐2) DPCs. Modified and reprinted with permission.^39^ Copyright 2010, American Chemical Society; (D) the sPLA2 hydrolysis of all‐transretinoic acid (ATRA) based sn‐2 mono‐embedded DPCs. Modified and reprinted with permission.^39^ Copyright 2010, American Chemical Society.

Compared with hydrophilic drugs, hydrophobic drugs are more dependent on nano‐carriers like liposome for delivery.^35^ In liposomal delivery systems, hydrophobic drugs are stabilized in the hydrophobic regions with aliphatic chains of the lipid‐bilayers.^36^ Therefore, using hydrophobic drugs to replace the original alkyl chains of phospholipids sounds reasonable. But, such DPCs based on hydrophilic drugs are reported as well. For example, Rosseto et al. reported a serial of non‐steroidal anti‐inflammatory drugs (NSAIDs) based sn‐2 mono‐embedded DPCs as sPLA2‐sensitive prodrugs (Figure 4A).^37^ Xiao et al. reported a 5‐fluorouracil‐derived DPC that adopts this structure with a special redox/pH sensitive linker.^40^ According to the results, this DPC can also participate in the assembly of liposomal structures and exhibit environmentally responsive release behavior. The other hydrophobic chain in the DPC can theoretically provide a hydrophobic domain that facilitates co‐assembly with traditional phospholipids.^41^

As mentioned before, the conjugation of hydrophobic drug and single‐chain lipids (lysophospholipids) can be directly done by a simple esterification reaction.^42^ Therefore, the sn‐2 mono‐embedded DPCs are quite classic and widely reported with a long history (Figure 4B).^37^, ^38^ Drug‐lysophospholipid conjugates are a typical sn‐2 mono‐embedded form. This type of sn‐2 mono‐embedded DPCs shows a great advantage because the ester bond in sn‐2 position is specifically sensitive to the sPLA2 enzyme, which is overexpressed in inflammation and tumor tissues.^18^, ^43^ The mechanism of sPLA2‐catalyzed release is shown in Figure 4C.^39^ X‐ray crystallography revealed that the catalytic mechanism of sPLA2 involves aspartic acid‐histidine dyad, a calcium‐binding site, and a water molecule serving as the nucleophile. Therefore, sn‐2 mono‐embedded DPCs could realize effective controlled release in inflammation and tumor tissues. Sn‐2 mono‐embedded DPCs could be easily generated from drugs with carboxy groups. But it must be noted that not all sn‐2 mono‐embedded DPCs from carboxylated molecules will be endowed with sPLA2 sensitivity, such as all‐trans retinoic acid (ATRA)‐lysophospholipid conjugates.^44^, ^45^, ^46^ This may be because the rigid structure of some drugs prevents enzymes from recognizing the sn‐2 ester bond. Computer simulation showed that adding a 6‐carbon link arm between the drug and the phospholipid greatly improved the enzymatic hydrolysis rate of sPLA_2_ (Figure 4D).^39^, ^47^

Compared with sn‐2 mono‐embedded DPCs, sn‐1 embedded reports are relatively rare. A sn‐1 modified example is a special phospholipid called pro anticancer ether lipids (ProAELs), which would accelerate the hydrolysis of the ester bond at the sn‐2 position after exposure to sPLA2 enzymes.^48^ It also found that while ether ester or thioether ester bonds were introduced to sn‐2 of ProAELs, their sPLA2 sensitivity was decreased dramatically.^49^ Similarly, some drugs or fluorescent molecules were indiscriminately conjugated to sn‐1 or sn‐2 by normal ester linkages, which usually not pursues for a rapid drug release. Besides of drug release strategy, some multi‐functional designs were also reported via sn‐1 or sn‐2 mono‐embedded DPCs because of the high synthetic efficiency. Different special molecules were conjugated to lysophospholipids for sorts of features especially the light‐sensitive phospholipids, which will be discussed particularly later in this review.^23^

The phospholipids used in their synthesis differ based on the position of the hydroxyl group at the sn‐1 or sn‐2 position, which determines the type of mono‐embedded DPCs produced. These can be obtained through specific hydrolysis by different esterases. For example, lysophospholipids with a hydroxyl group at the sn‐2 position can be generated by incubating phospholipids with sPLA2. Using these lysophospholipids and active molecules as reactants, mono‐embedded (sn‐2) DPCs can be synthesized.

### 3.3 Bis‐embedded (sn‐1 and sn‐2) DPCs

**FIGURE 5:**
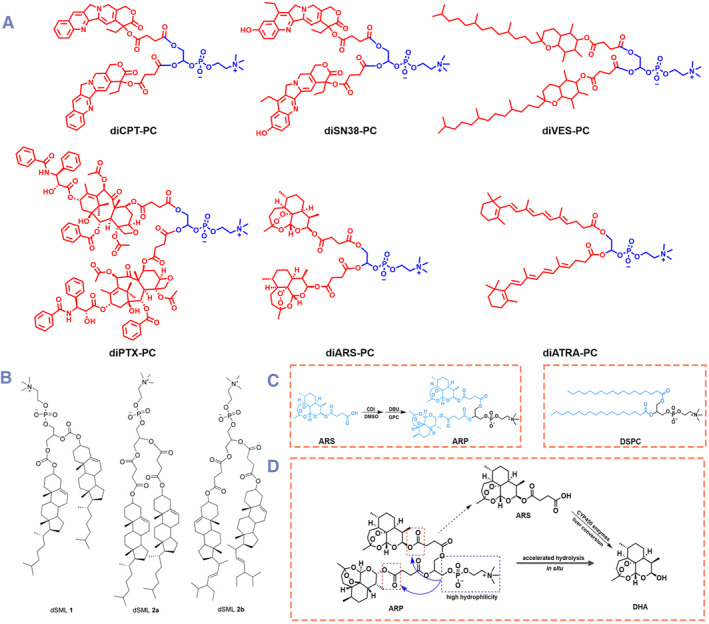
Bis‐embedded (sn‐1 and sn‐2) DPCs: (A) hydrophobic drugs (red color) were conjugated to GPC (blue color) under specific heterogeneous reaction conditions; (B) bis‐sterol embedded DPCs (named as disterol‐modified phospholipids). Modified and reprinted with permission.^12^ Copyright 2009, John Wiley and Sons; (C, D) synthesis of artesunate based bis‐embedded (sn‐1 and sn‐2) DPC and the hydrolysis mechanism. Modified and reprinted under terms of the CC‐BY license.^51^ Copyright 2022, The Authors, published by American Association for the Advancement of Science.

Compared with mono‐embedded DPCs, bis‐embedded (sn‐1 and sn‐2) DPCs can obtain a more symmetrical structure, and much higher drug loading as well. Different from the high yield of mono‐embedded DPCs, bis‐embedded DPCs were often restricted by the poor synthetic efficiency with common catalytic system and separation technology. Therefore, the early reports of bis‐embedded phospholipids are not applicable for drug delivery. Regen et al. synthesized bis‐lipoic acid embedded DPCs as cross‐linkable phospholipid mimics to be used in physical property research of lipid‐bilayers.^50^ With the development of synthesis and separation techniques, more and more bis‐embedded DPCs have emerged recently (Figure 5A). For example, Szoka et al. reported a category of bis‐sterol embedded DPCs (named as disterol‐modified phospholipids), which could directly replace traditional phospholipids to prepare liposomes (dSML) for encapsulating doxorubicin (Figure 5B).^12^ Doxorubicin loaded dSML showed similar efficacy with commercial Doxil^TM^ during in vivo anti‐cancer evaluation.

Most hydrophobic drugs could be conjugated to glycerophosphatidylcholine (GPC) under specific heterogeneous reaction conditions, such as N, N′‐carbonyl diimidazole (CDI) and 1, 8‐diazadicyclic [5.4.0] undecan‐7‐ene (DBU) catalytic system.^52^ Li et al. reported a large collection of such bis‐embedded (sn‐1 and sn‐2) DPCs, and there have been over 10 hydrophobic drugs were applied in this heterogeneous reaction with GPC, such as chlorambucil,^52^ paclitaxel,^53^ camptothecin,^54^ 7‐ethyl‐10‐hydroxycamptothecin (SN‐38),^55^ podophyllotoxin,^56^ all‐trans retinoic acid,^57^ bexarotene,^58^ vitamin E succinate^59^ and artesunate.^60^ Compared with mono‐embedded DPCs, bis‐embedded DPCs have more symmetrical structures, which would greatly improve the assembly stability. More importantly, these two drug molecules embedded conjugates give the following assembly nearly double drug loading capacities. Based on these, bis‐embedded (sn‐1 and sn‐2) DPCs seem much more appealing than mono‐embedded conjugates. Moreover, the hydrolysis reaction of bis‐embedded (sn‐1 and sn‐2) DPCs is effective as well to release the parent drugs (Figure 5C,D).^51^

Moreover, a similar CDI/DBU catalytic system could be used to design new phospholipid‐based drugs. Carboxylic acids with pharmaceutical active groups, such as thioethers and disulfide bonds, could be easily conjugated to GPC.^61^, ^62^, ^63^ Although these conjugates are not exactly the prodrugs, they greatly enriched the design ideas of DPCs.

The advantages of DPCs over other drug delivery systems can be summarized as follows: DPCs are amphipathic small molecules with well‐defined chemical structures, unlike drug‐polymer conjugates, which allows for better control over synthesis, characterization, and clearer metabolic pathways. Their chemical structure closely resembles traditional phospholipids, exhibiting similar physical properties. This similarity enables the nano‐assembly of DPCs using established liposome processes, reducing the need for extensive process development. Additionally, the phospholipid‐like bilayer structure of DPCs has high homology with cell membranes, improving safety and facilitating membrane fusion. This unique structure also supports hybridization with cellular components, expanding the range of potential applications.

## 4 STIMULI‐TRIGGERED DESIGN OF DPCS

### 4.1 sPLA2‐triggered DPCs

**FIGURE 6:**
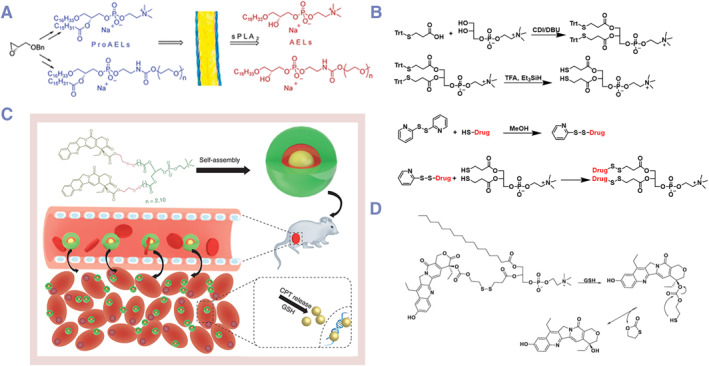
Examples of stimuli‐triggered DPCs: (A) lipid‐soluble anti‐tumor drugs, as antitumor ether lipids (AELs), are conjugated to sn‐2 position of phospholipids as sPLA2‐triggered prodrugs of antitumor ether lipids (proAELs). Modified and reprinted with permission.^69^ Copyright 2004, American Chemical Society; (B) a synthesis route of disulfide bonds‐based DPCs. Modified and reprinted with permission.^62^ Copyright 2019, American Chemical Society; (C, D) redox‐triggered camptothecin‐based DPCs for sensitive release. Modified and reprinted with permission.^70^ Copyright 2019, Taylor and Francis.

In all the studies of responsive DPCs, the drug release control responsive to secretory phospholipase A2 (sPLA2) was the first to be reported.^49^ sPLA2 is a class of enzymes that belong to the phospholipase family.^64^ These enzymes play a crucial role in the body by catalyzing the hydrolysis of phospholipids to produce free fatty acids and lysophospholipids.^65^ For example, lysophospholipids can act as key recruiting molecules to activate the chemotaxis of phagocytes to the lesion site.^66^ High expression of sPLA2 can be observed under various physiological and pathological conditions, including tumor, inflammatory processes, infections, cardiovascular diseases and metabolic diseases.^67^, ^68^ A lot of lipid‐soluble anti‐tumor drugs, as antitumor ether lipids (AELs), are conjugated to sn‐2 position of phospholipids as sPLA2‐triggered prodrugs of antitumor ether lipids (proAELs) (Figure 6A).^69^

Besides, the significance of high sPLA2 expression mainly lies in its role in regulating inflammation and immune responses.^71^ While they play a crucial role in defending the body, abnormally high sPLA2 activity is also associated with various disease states, suggesting that they could be potential therapeutic targets. Therefore, sPLA2 sensitivity has been researched for the treatment of inflammation‐related diseases and other conditions.

Generally, sPLA2 selectively hydrolyzes the ester bond at the sn‐2 position of phospholipids, thus producing lysophospholipids and the corresponding fatty acids. Therefore, if the sn‐2 position is linked to an active drug molecule, sPLA2 will directly induce the release of the active molecule. At the same time, the phospholipid‐related assembly structure will be destroyed, and physically loaded drugs will also be released synchronously. Although phospholipid liposomes generally have natural selectivity for sPLA2, research also shows that special structural designs can enhance the selectivity for sPLA2. For example, the truncated oxidized phosphatidylcholine would be more sensitive to be hydrolyzed by sPLA2 compared to normal phospholipids such as 1,2‐dioleoyl‐sn‐glycero‐3‐phosphocholine (DOPC).^72^ Therefore, designing DPCs and liposomal carriers that respond to the high expression of sPLA2 in certain pathological environments is of practical significance. Besides, as mentioned above, the rigid structure of some drugs prevents the enzyme from recognizing the sn‐2 ester bond.^44^, ^45^, ^46^ Therefore, it could be used to explain why a longer linker between the drug and the phospholipid could increase sPLA2 enzymatic hydrolysis. In addition to truncated oxidized phosphatidylcholine, various other sPLA2 enzyme‐sensitive phospholipids with different mechanisms were reported. For example, Andresen et al. synthesized a phospholipid where the sn‐1 position has an unhydrolyzable ether bond and the sn‐2 position has a normal ester bond.^69^

Besides, because the sPLA2 enzyme has a high specificity for recognizing anionic ester membranes, the responsiveness to sPLA2 can be enhanced by preparing negatively charged phospholipids and shortening the length of their fatty acids.^73^ A typical example of such enzyme‐sensitive phospholipid is distearoyl phosphatidylglycerol (DSPG). Currently, the sPLA2 enzyme‐sensitive liposome loaded with cisplatin (brand name LiPlaCis^®^), based on DSPG, has passed Phase I clinical trials for advanced solid tumors.^73^ However, the development path of LiPlaCis^®^ has not been smooth as its early clinical trials were once suspended due to safety concerns with the formulation. The initial formulation of LiPlaCis^®^ was too unstable, leading to high concentrations of free cisplatin that caused renal toxicity. The mechanism of this side effect does not seem to be related to the concentration of sPLA2 enzyme in normal tissues and remains unclear to this day. Nonetheless, this did not halt the development of sPLA2 enzyme‐sensitive liposomes; by modifying the formulation and supplementing with the injection of chlorpheniramine and dexamethasone, these side effects can be significantly alleviated.^73^

### 4.2 Redox‐triggered DPCs

Redox‐responsive strategies are specifically tailored to the particular redox states which significantly differ between normal physiological environments and disease states (such as cancer and inflammation).^74^ The microenvironment of cancer cells and certain inflammatory regions often exhibit a higher level of reductiveness (for example, higher levels of glutathione (GSH) and/or lower levels of oxidants), providing a foundation for designing carriers that can release drugs specifically under these conditions.^75^

Many structures have been reported to exhibit redox responsiveness and are utilized in the design of DPCs. Typical structures include disulfide,^76^ thioether,^77^ diselenide,^78^ selenoether^79^ and thioketal bonds.^80^ Generally, these redox‐sensitive structures will break, leading to the sensitive release of the drug. Alternatively, they induce the disassembly of the structure, indirectly accelerating the hydrolysis of ester bonds. Among them, the design of disulfide and thioether bonds is the most classic.^61^ Disulfide bonds are one of the most widely used redox‐responsive linkers which can reversibly break and re‐form under reducing conditions.^61^ Additionally, they can increase the hydrophilicity of drug systems, promoting the hydrolysis of adjacent chemical bonds (such as ester bonds) and the release of free drugs. In contrast, thioether bonds primarily exhibit oxidative responsiveness.^62^ It was found that they can be oxidized into hydrophilic sulfoxides or sulfones, inducing the disruption of assembled structures through hydrophilic‐hydrophobic transitions, while also enhancing the hydrolysis of neighboring ester bonds.^62^

Generally, thioether and disulfide bonds are designed as linkers between the parent drug molecules and phospholipids at the sn‐1 or sn‐2 positions. The hydroxyl group of the original drug molecule is first extended through a thiol‐containing anhydride structure, and then by esterification reaction with lysophospholipids, mono‐embedded (sn‐1 or sn‐2) DPCs can be easily synthesized.^70^ However, the synthesis of bis‐embedded (sn‐1 and sn‐2) DPCs can be much more complex. In order to achieve efficient heterogeneous esterification reactions, harsh reaction conditions (CDI/DBU catalytic system) can destroy related structures, especially disulfide bonds. To address this issue, a unique thiol‐exchange reaction has been designed, involving the initial synthesis of bis‐embedded (sn‐1 and sn‐2) DPCs with thiol‐protected structures (Figure 6B).^61^, ^70^ These redox‐triggered DPCs usually demonstrate better tumor and inflammation‐responsive release compared to ordinary DPCs (Figure 6C,D).

### 4.3 pH‐triggered DPCs

pH response is another important type of smart responsive prodrug design, which is particularly suitable for targeting specific diseases such as cancer or infected areas, where the pH values often differ from those of healthy tissues.^81^ The tumor environment is the most frequently reported application scenario for pH‐responsive prodrugs.^82^ Many tumors are slightly more acidic than normal tissues. pH‐sensitive drug delivery systems can exploit this feature to directly release chemotherapeutic drugs at the tumor site, minimizing systemic toxicity.^83^ Drugs can be connected to carriers through chemical bonds that break under specific pH conditions. Many chemical structures are pH‐sensitive, including hydrazone bonds, ester bonds, sulfonamide bonds, and amino acid derivatives (such as cysteine derivatives).^84^ Among them, the design of the hydrazone bond is the most classic, which is stable at neutral pH but breaks under acidic conditions.

Hydrazone bonds are widely used in the design of polymer conjugate prodrugs, whereas the design of DPCs is relatively less common.^85^ The selected parent drugs often primarily include paclitaxel (PTX) and doxorubicin (DOX).^86^ Although hydrazone linkage structures are rarely used directly in DPCs, these structures are applied in the design of pH‐sensitive phospholipid derivatives. In this context, PEGs are connected to DSPE via hydrazone bonds.^87^ The resulting PEG lipids can assist in the removal of the hydrophilic outer shell of the assembled liposomes in acidic environments, enhancing cellular uptake.^88^ Such designs indirectly aid the practical application of DPC assemblies. Although designs for DPCs that utilize hydrazone bonds are currently scarce, the concept is theoretically viable. This is particularly true for strategies that involve coupling DOX to the polar head of phospholipids through hydrazone bonds.

In addition to hydrazone bonds, ortho esters, vinyl ether, and α,α‐difluoro ester were also reported to have pH responsiveness and can be applied in the design of DPCs.^89^ For example, Szoka et al. reported a pH‐sensitive phospholipid small molecule achieved by introducing an ortho ester linker that is sensitive to acid cleavage in one of the hydrophobic tails of the phospholipid.^90^ In acidic media, one of the hydrophobic chains of the phospholipid can efficiently cleave, thus destabilizing the phospholipid assembly. Luo et al. designed an optimized pH‐sensitive phospholipid using pentaerythritol as a backbone. An intermediate group of trialkoxybenzene (1,3,5‐trialkoxybenzene) was added between the ortho ester structure and the phospholipid head.^91^ The addition of the spacer group further enhances acid sensitivity. Clausen et al. reported that phospholipids containing a difluoroester (α,α‐difluoro ester) structure tend to accelerate hydrolysis under acidic conditions.^92^ The closer the difluoroester structure is to the phospholipid head, the more pronounced this tendency for accelerated hydrolysis. All these structures could be used for the design of pH‐triggered DPCs.

### 4.4 Light‐triggered DPCs

Photosensitive liposomes are primarily achieved through the design of sensitive phospholipids.^93^ Unlike other sensitive phospholipids, the types and mechanisms of photosensitive phospholipids are relatively complex, with some even involving multiple sensitivity mechanisms.^94^ In simple terms, the mechanisms of photosensitive phospholipid liposomes can be divided into photosensitive cleavage,^95^ photosensitive polymerization,^95^ photosensitive isomerization,^96^ and covalently coupled photosensitizer activation.^97^ Photosensitive cleavage phospholipids utilize special structures within the phospholipid that are prone to break under certain wavelength of light. Among them, the ortho‐nitrobenzyl (o‐NB) group is one of the most common structures for photosensitive cleavage.^98^ For example, Bowman et al. prepared photosensitive cleavage phospholipids by connecting hydrophobic chains containing the o‐NB structure to the phospholipid structure using in situ click chemistry.^99^ Under UV light exposure at 320–390 nm, the carbonate next to the o‐NB structure in these phospholipids efficiently cleaves, leading to the disruption of the phospholipid's hydrophobic‐hydrophilic structure. Liposomes assembled from these phospholipids also exhibit significant photosensitivity under the same wavelength of UV light, rapidly releasing the encapsulated drug for sensitive release. According to the cleavable feature, the o‐NB group could be used for light‐triggered DPCs design.

Different from cleavable DPCs, photosensitizer‐phospholipid conjugates could be regarded as another light‐triggered DPCs, which could respond directly to light conditions. These photosensitizers undergo photochemical or photophysical changes upon illumination such as photoisomerization, photo‐oxidation reactions, or photothermal conversions.^100^ Numerous photosensitizers have been reported for the construction of photosensitive drug carriers, such as methylene blue, indocyanine green, porphyrins, and phthalocyanine derivatives.^101^ In the field of photosensitizer‐phospholipid conjugates, the most notable work comes from Lovell group, which has extensively reported on porphyrin‐phospholipid (PoP) systems.^23^, ^102^ In PoP structures, the fatty acid at the sn‐2 position of the phospholipid is replaced by a porphyrin derivative. Liposomes incorporating PoP (molar ratio: DSPC 60%, cholesterol 35%, PEG‐lipid 5%, and PoP 10%) exhibit changes in permeability under near‐infrared light, with their loaded drugs being nearly completely released within 3 min. PoP can be used not only to prepare photosensitive liposomes but also to fabricate multimodal imaging systems by doping with other luminescent materials such as upconversion luminescent materials.^103^

### 4.5 Shear stress‐triggered DPCs

Certain cardiovascular diseases exhibit significant differences in shear stress at the lesion sites. For example, arteriosclerosis leads to arterial narrowing, resulting in a significant increase in shear stress at the lesion sites compared to healthy vessels.^104^ This rheological property change can also be targeted for the design of lipid‐based drug delivery systems. One of the most notable examples is reported by the Saxer group, which utilized a lipid with amide bonds (1,3‐diaminophospholipid, Pad‐PC‐Pad) to prepare shear‐sensitive liposomes.^105^, ^106^, ^107^ Under cryo‐transmission electron microscopy, these liposomes exhibit a unique lentil‐like shape. They remain stable under the normal stress conditions of healthy vessels but release their drug payload extensively when subjected to the high stress of constricted vessels.^107^ This approach offers a unique strategy for treating cardiovascular diseases such as arteriosclerosis. If the alkyl chains were replaced with drug‐synthesizing DPCs, such DPCs may also have similar stress responsiveness, inducing disintegration of the assembly and enhancing the cellular uptake rate of the disintegrated free DPCs. At present, this kind of design is not directly reflected in DPCs, but the reported structure has guiding significance for the future design of stress‐responsive DPCs.

DPCs are primarily designed for intracellular release and function although extracellular responsive release is also common. Regardless of release location, DPCs typically target intracellular proteins. While designs targeting membrane proteins exist, they are relatively rare. As prodrugs, DPCs undergo efficient intracellular degradation to release active components, maximizing drug stability and efficacy. Conventional designs focus on enhancing intracellular release, especially in tumor therapy, where DPCs are engineered to respond to tumor‐specific conditions like pH and redox environments.^75^ However, some DPCs are designed to respond to extracellular environments or non‐specific conditions, as the release of lipophilic small molecules generally does not hinder rapid cellular uptake. For example, photosensitive and shear‐sensitive DPCs function independently of the intracellular environment, and their efficacy is unaffected by whether release occurs inside or outside the cell.

DPCs based assemblies primarily enter cells through endocytosis, similar to traditional nanoparticles. For nano‐scale assemblies, free diffusion and microchannels are ineffective for cellular uptake, but endocytosis enables efficient internalization. Membrane protein families, such as TLRs, recognize and facilitate nanoparticle uptake in most cells.^26^ DPCs resemble liposomes, with polar phospholipid surfaces facilitating similar uptake pathways. Therefore, there is little doubt about the cellular uptake of DPCs, and experimental data support this mechanism. The endocytosis of DPCs and liposomes is also influenced by cholesterol incorporation as a simple phospholipid structure alone is insufficient for efficient uptake. Additionally, a small percentage of DPCs may enter cells via membrane fusion.

## 5 APPLICATIONS OF PDCS AND PDC NANO‐ASSEMBLIES

### 5.1 Anti‐tumor applications

Antitumor therapy is the most extensively documented application for PDCs. Many antitumor active molecules, characterized by their benzene ring conjugated structures and inherently low solubility, are covalently conjugated to phospholipid structures and self‐assemble into nanomedicines.^108^ PDCs significantly enhance the solubility of drugs that are otherwise poorly soluble by forming amphiphilic structures with drugs, facilitating better dispersion and absorption within the body. Additionally, due to the affinity of phospholipid molecules for biological membranes, PDCs can penetrate cell membranes more effectively. This characteristic enables the drug to directly target intracellular sites, thus amplifying therapeutic outcomes.

Moreover, most PDCs nano‐assemblies, ranging in size from 100–200 nm, exhibit passive tumor targeting capabilities due to the Enhanced Permeability and Retention (EPR) effect.^109^, ^110^ Furthermore, by targeting the unique microenvironment of tumor tissues (such as areas with low pH, specific redox conditions, or enzyme expressions), PDCs with responsive structures selectively accumulate in tumor cells or specific tissues, achieving precise site‐specific drug release. For example, the PLA2 that specifically hydrolyzes the sn‐2 position, as previously mentioned, is an enzyme highly expressed in tumors, making the ester bonds at the sn‐2 position very susceptible to hydrolysis.^111^ Therefore, mono‐embedded (sn‐2) DPCs are mostly common for anti‐tumor applications. Based on this, other sensitive linkers will be further applied to modify the connection between the active molecules and the sn‐2 structure.

Bis‐embedded (sn‐1 and sn‐2) DPCs also show significant accelerated drug release and both ester bonds in sn‐1 and sn‐2 would be hydrolyzed under tumor environments.^55^ Compared with mono‐embedded DPCs, Bis‐embedded DPCs have more symmetrical structures, which contribute to their assembly stability. When the hydrolysis of the sn‐2 ester bond occurs, this stable structure is rapidly destroyed, resulting in rapid disintegration of the bilayer. The increase in molecular hydrophilicity caused by the hydrolysis of the sn‐2 ester bond will accelerate the hydrolysis of the sn‐1 position, especially in the tumor environment. Therefore, we can find that many bis‐embedded DPCs also show very decent anti‐tumor activity.

Additionally, PDCs can serve as carriers for both lipid‐soluble and water‐soluble drugs, facilitating combination therapies.^112^ Vesicles formed from DPCs that incorporate low‐rigidity lipid active molecules generally demonstrate enhanced drug loading capabilities.^113^ This improvement may stem from their lower phase transition temperatures and increased molecular chain fluidity. Complex assembly systems, such as nano‐fibrous structures, can also arise if there are specific interactions between the DPCs and the drugs they carry. The phenyl ring *π*‐π conjugated structure, often found in anti‐tumor agents, is especially likely to promote such phenomena.^114^, ^115^

Currently, the majority of DPCs employed in anti‐tumor applications are built on a glycerol framework. Their anti‐tumor efficacy primarily derives from substituting hydrophobic tails with anti‐tumor active molecules like camptothecin and paclitaxel.^53^, ^55^ These DPCs exhibit a classical prodrug mechanism. The active sites were sealed with ester bonds, significantly enhancing the stability of the active molecules. Their efficacy is reactivated through hydrolysis in specific environments. The use of DPCs with non‐glycerol backbones for constructing anti‐tumor vesicles was also reported. For instance, a phospholipid‐docetaxel conjugate can be structured using lithocholic acid as the backbone.^116^ These DPCs also retained their vesicle self‐assembly capabilities. By combining with polyethylene glycolylated lipid‐dexamethasone conjugates, they can self‐assemble into chimeric nano‐micelles smaller than 100 nm. These nano‐micelles, by influencing the prostaglandin pathway, can improve the immunosuppressive inflammatory tumor microenvironment, slow tumor progression, and enhance survival rates in mice.

### 5.2 Anti‐inflammatory applications

In addition to their application in the field of anti‐tumor treatments, another major application of DPCs is in anti‐inflammatory treatments. Similar to tumor tissues, overexpression of sPLA2 enzymes is also observed in inflamed tissues. Therefore, DPCs naturally act as sensitive structures at sites of inflammation, and even the most basic DPCs display a certain degree of inflammatory responsiveness. Clearly, targeting the overexpression of sPLA2 enzymes at inflammatory sites by substituting drugs at the sn‐2 position will exhibit pronounced inflammatory responsiveness. For example, non‐steroidal anti‐inflammatory drugs are covalently conjugated to the phospholipid sn‐2 position through a linker arm for targeted therapy for inflammatory bowel disease (IBD).^17^, ^117^ DPCs can be administered in various ways, including intravenously, orally, or intracolonically, all achieving the pharmacological effects of the parent drugs. Additionally, the length of the linker arm significantly affects the activity of sn‐2 hydrolysis. Experimental results from multiple research teams all showed that DPCs with longer linker arms exhibited higher rates of prodrug activation in response to sPLA2 enzymes.

**FIGURE 7:**
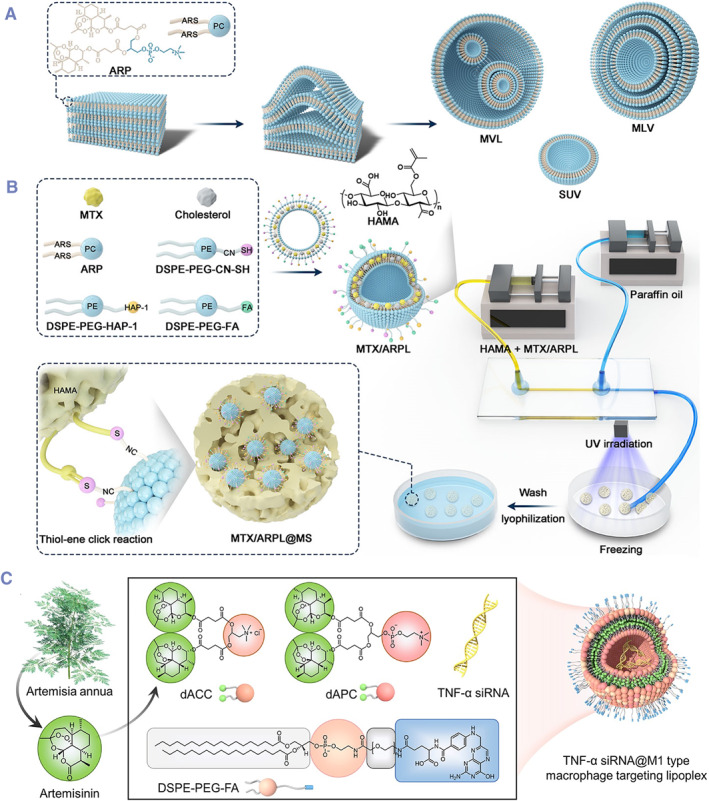
Dimer artesunate‐phospholipid conjugates (diART‐PC): (A) diART‐PC could be assembled into liposomes using traditional lipid preparation techniques. Modified and reprinted under terms of the CC‐BY license.^51^ Copyright 2022, The Authors, published by American Association for the Advancement of Science; (B) diART‐PC liposomes could be encapsulated into microspheres as injectable micro‐complexes for intravenous injection. Modified and reprinted under terms of the CC‐BY license.^51^ Copyright 2022, The Authors, published by American Association for the Advancement of Science; (C) dimer cationic artemisinin derivative was added to the assemblies to expand its gene carrier function, which was used to co‐delivery TNF‐α siRNA for exploring gene/chemotherapeutic agent combination RA therapy. Modified and reprinted with permission.^120^ Copyright 2022, John Wiley and Sons.

Although non‐steroidal anti‐inflammatory drugs (NSAIDs) can be formulated into nanoparticles via direct physical loading, this method has been widely reported in the field of disease treatment. Beyond the response to sPLA2 enzymes, strategies through drug conjugation still hold significant advantages such as increased water solubility, high drug loading, and enhanced stability. A typical example is methotrexate, which has very low water solubility, however stable nanoparticle formulations can be obtained through DPC strategies.^118^ Moreover, some anti‐inflammatory active molecules are highly unstable and can degrade rapidly even at room temperature. This low stability leads to drawbacks such as short half‐lives. A typical example is artemisinin and its derivatives. Li et al. reported the systemic injection of self‐assembling complexes of dimer artesunate‐phospholipid conjugates (diART‐PC) for the treatment of rheumatoid arthritis (RA).^119^ The liposomes were prepared using traditional lipid preparation techniques and obtained uniform size through high‐pressure shearing (Figure 7A). After intravenous injection through the tail vein, the RA model mice exhibited significantly improved disease symptoms, and inflammatory markers in the blood were noticeably reduced. Besides, diART‐PC assemblies were used as drug carriers for MTX loading as co‐delivery systems (Figure 7B).^51^ To enhance the in situ intra‐articular injection, hydrogel microspheres were used to encapsulate co‐delivery assemblies for a prolonged sustainable release.^121^ Another dimer cationic artemisinin derivative was added to the assemblies to expand its gene carrier function, which was used to co‐delivery TNF‐α siRNA for exploring gene/chemotherapeutic agent combination RA therapy (Figure 7C).^120^

### 5.3 Antiviral applications

DPCs are also an efficient choice for antiviral prodrugs. The unique phospholipid structure provides the prodrugs with significantly improved cell membrane penetration, which is crucial in the antiviral field. For example, in the case of the SARS‐CoV‐2 virus, Carlin et al. reported a series of remdesivir‐based DPCs aimed at enhancing the efficacy of oral administration.^122^ These DPCs partially retained the structure of traditional DPCs. Since remdesivir is a hydrophilic molecule, in the related DPC structures, remdesivir was substituted at the polar end, while the hydrophobic end was modified by replacing one lipid with a benzene ring structure to enhance recognition by phospholipase C.

### 5.4 Vaccine applications

DPCs have also been reported to serve as adjuvants in the vaccine field, enhancing the delivery of antigens to immune cells.^123^ When used in vaccines, DPCs offer several significant advantages that could potentially improve both the efficacy and the safety profile of the vaccines.^124^ Being generally biocompatible and biodegradable, DPCs reduce the risk of toxicity and adverse reactions that are often associated with other synthetic delivery vehicles. Additionally, DPCs can be engineered to target specific cells or tissues, thereby improving the specificity of the immune response.^125^ This targeting capability is particularly valuable in cancer vaccines where directly targeting tumor cells can significantly boost efficacy. Furthermore, the physical and chemical properties of DPCs, such as size, charge, and surface characteristics, can be customized during synthesis to optimize their interaction with the immune system, enhancing the overall effectiveness of the vaccine.

By directly conjugating bioactive molecules with phospholipids, a biomimetic effect can be achieved to enhance the immunogenicity of vaccines. For example, Liu et al. reported a vaccine strategy involving the self‐assembly of amphiphilic DPCs synthesized from 1,2‐dioleoyl‐sn‐glycero‐3‐phosphoethanolamine (DOPE) and human unmethylated oligodeoxynucleotides (CpG ODN), a Toll‐like receptor agonist. This structure induces antigen cross‐presentation and initiates CD8^+^ T‐cell responses, thereby providing effective initial antigen stimulation and sustained long‐term immune effects.^126^ This approach utilizes a biomimetic effect by mimicking natural processes within biological systems to enhance the overall immune response of vaccines.

Besides, special molecular affinities can also be applied to the binding of DPCs with antigens, significantly enhancing the immunogenicity of the associated vaccines. A classic example in this field is the cobalt‐porphyrin phospholipid (CoPoP). As mentioned above, CoPoP itself is a type of photosensitive DPCs.^23^, ^102^, ^127^ However, due to the cobalt porphyrin structure of CoPoP having affinity with the polyhistidine‐tag (his‐tag), which is the widely used tag protein for protein purification, the assembled CoPoP‐based liposomes demonstrated significantly stabilized loading of recombinant proteins or polypeptides containing histidine residues. Initially, CoPoP was utilized for the construction of recombinant malaria vaccine nanoparticulation. Through affinity adsorption, the soluble recombinant antigen (Pvs25) was modified on the surface of colloidal particles to enhance its immunogenicity.^128^ The following studies found that the CoPoP assembled adjuvant is a universal antigen carrier as long as the antigen contains a histidine sequences.^129^ Therefore, the application of this DPCs vaccine carrier has been greatly expanded and is now used for acquired immunity of different pathogens such as cancer, human immunodeficiency virus (HIV),^130^, ^131^ coronavirus 2 (SARS‐CoV‐2)^132^, ^133^, ^134^ and influenza virus.^135^, ^136^, ^137^

### 5.5 Anti‐malarial applications

In addition to CoPoP‐based anti‐malarial vaccines, the conjugation of active molecules can directly achieve anti‐malarial efficacy and application. Based on the natural anti‐malarial activity of artemisinin and its derivatives, artemisinin‐based DPCs have been developed to enhance their efficacy and expand their application approach.^60^, ^138^ By conjugating artesunate with glycerophosphocholine through esterification reaction, the rapidly constructed diART‐PC possesses a chemical structure similar to traditional phospholipids and can efficiently form liposome assemblies with bilayer structures. Unexpectedly, the diART‐PC assemblies have shown a certain degree of capability to overcome *Kelch 13* mutant artemisinin resistance. The *Kelch 13* mutant of the *Plasmodium falciparum* malaria parasite results in notable artemisinin tolerance during its ring stage, significantly reducing the efficacy of artemisinin‐based drugs. Since these drugs typically have a very short half‐life and act quickly, this leads to reduced effectiveness against mixed‐stage *Plasmodium falciparum* infections and an increased rate of recurrence. Therefore, the diART‐PC assemblies significantly improve the stability of artemisinin‐based drugs by enabling controlled release in the body. This resulted in a markedly increased half‐life for diART‐PC, thereby achieving a more effective killing of mixed‐stage *Plasmodium falciparum*. This enhancement in anti‐malarial efficacy directly stems from the unique structure and assembly capabilities of the DPCs.

### 5.6 Anti‐epileptic applications

DPCs are also frequently used in the treatment of epilepsy. According to the literature, the celecoxib–phospholipid conjugate exhibits potential anticonvulsant properties under the action of the sPLA2 enzyme.^139^ Celecoxib, a non‐steroidal anti‐inflammatory drug, inhibits cyclooxygenase‐2 (COX‐2), which is reported to play a significant role in neuroinflammation, particularly in the progression of epilepsy. COX‐2, an enzyme produced during inflammation, plays a crucial role in synthesizing prostaglandins (inflammatory mediators). In the context of neuroinflammation and epilepsy, overactivated COX‐2 can enhance inflammatory responses, potentially exacerbating the occurrence and progression of epilepsy. By conjugating celecoxib with phospholipids, the bioavailability and stability of the drug can be improved while potentially reducing its systemic side effects. Since phospholipids are components of cell membranes, this conjugate may be more readily absorbed by cells and release the active ingredient where needed such as at inflammation sites.^140^

In addition to celecoxib, valproic acid is a pivotal antiepileptic drug. It functions by increasing the levels of gamma‐aminobutyric acid (GABA) in the brain which helps to inhibit nerve transmission and thus reduce seizure activity.^141^ Valproic acid also modulates ion channel activity, further contributing to its anticonvulsant effects. This drug is effective in treating various types of seizures and is often utilized in patients who have not responded well to other treatments. However, it is crucial to monitor potential side effects, such as liver toxicity and teratogenic effects, especially when used over long periods. By conjugating valproic acid with phospholipids to create DPCs (DP‐VPA),^142^, ^143^ its bioavailability can be enhanced and liver toxicity reduced. This approach leverages the biocompatibility and bioavailability advantages of phospholipids to improve the delivery and efficacy of valproic acid, potentially leading to better therapeutic outcomes and diminished side effects.

### 5.7 Other applications

Due to the high flexibility in the selection of drug components in DPCs, the application scenarios of DPCs can be effectively expanded based on different drug choices. This openness allows DPCs to select the most suitable drug components according to specific diseases or therapeutic needs, thereby addressing various medical issues in a targeted manner.

Additionally, DPCs can also be directly used as excipients for nanomedicines, such as those formed by the conjugation of *α*‐lipoic acid with phospholipids. Due to the natural antioxidant properties of *α*‐lipoic acid, its conjugates can serve as broad‐spectrum formulation excipients for the preparation of liposomes, enabling the liposomes themselves to possess antioxidant functionality.^144^ Similarly, DPCs with thioether structures are not only active pharmaceuticals but also can be directly considered as excipients with oxidative responsiveness. These excipients are particularly valuable in formulations where the oxidative environment can trigger specific responses, enhancing the delivery and efficacy of the therapeutic agents encapsulated within. Meanwhile, such *α*‐lipoic acid containing DPCs were also reported to stabilize gold nanoparticles using the thiol‐gold coordination.^145^

In addition to traditional drugs, the lipid conjugate formed by covalently attaching fluorescent carbon dots (C‐dots) to phospholipids serves as a versatile and effective tool for studying bilayer dynamics.^146^ These special DPCs are used to elucidate the effects of various active molecules on the fluidity of phospholipid bilayers. Even, small interfering RNA (siRNA) is directly conjugated to phospholipids through chemical bonds, allowing the hydrophilic siRNA and the hydrophobic phospholipid tails to self‐assemble, overcoming the stiff backbone structure of siRNA and enhancing the loading efficiency of siRNA.^147^

## 6 CONCLUSIONS

This review highlights the innovative design and diverse applications of Drug‐Phospholipid Conjugates (DPCs) in drug delivery systems. DPCs, by integrating drug molecules with phospholipid structures, address the challenges of low drug loading and leakage seen in traditional liposomes. These conjugates maintain the self‐assembly properties of phospholipids and enable targeted drug release in specific pathological conditions, thereby enhancing efficacy and minimizing side effects. DPCs are designed to replace traditional phospholipids in liposomal structures, offering enhanced environmental sensitivity, high drug loading, and improved stability. They can respond precisely to various drug release environments by incorporating drug molecules into different structural components of phospholipids, allowing tailored release mechanisms such as redox or photosensitivity. The exploration of DPCs continues with potential expanded applications across multiple fields due to their unique structural properties.

## 7 FUTURE PERSPECTIVES

The research on DPCs will not stop here. Due to their other unique structural features, DPCs still hold great potential for expanded applications in many fields. The future application prospects of DPCs include but are not limited to the following:

1. Firstly, DPCs are used in the construction of functionalized/intelligent drug delivery systems. Due to their structure similar to phospholipids, DPCs are very suitable for constructing various hybrid liposomes and vesicles. The high affinity at the molecular level is not only demonstrated between DPCs and natural phospholipids but also among different DPCs as well as between DPCs and hydrophobic interfaces. Alternatively, specially modified DPCs can also interact with other substances through different covalent and non‐covalent forces. For example, CoPoP interacts with His‐tagged proteins, and thioctic acid‐phospholipid conjugates with gold nanoparticles.^145^
2. Furthermore, the application of DPCs in gene delivery is increasingly gaining attention. The rise of lipid nanoparticle (LNP) technology has led to a revolutionary advancement in the clinical translation of mRNA‐based gene therapy techniques. Thanks to the substitution of traditional phospholipids and lipids with DPCs, the development of new DPCs provides unlimited possibilities for the future functional expansion of LNP. Currently, there are not many cases of using DPCs within the LNP system, mainly because the traditional phospholipid/lipid‐based LNP technology is not yet mature, and phospholipids themselves do not constitute a high proportion in LNP formulations. However, phospholipid components are crucial in the formulation of LNPs, and the impact of their substitution with DPCs on LNP assemblies is yet to be determined.
3. In addition to their potential use in cell membrane fusion due to their high structural similarity to phospholipids, DPCs can also facilitate the embedding of drug molecules or other active substances as part of the membrane structure.^148^ This is done through techniques such as membrane fusion proteins and virus induction. A typical example of this application is the use of carbon dot‐phospholipid conjugates to study the dynamic characteristics of phospholipid bilayers. Indeed, the capabilities of DPCs extend beyond just real‐time tracing of the physical state of phospholipid bilayers. The membrane fusion properties of DPCs can also be utilized to regulate the microenvironment structures of cellular membranes and organelle membranes. This ability to manipulate membrane architecture at a micro‐level offers significant potential for targeted therapeutic interventions and detailed cellular studies.^149^ With DPCs, similar biomimetic liposomal strategies would be generated as well.^150^, ^151^
4. In addition to artificially synthesized DPCs, we can also explore the natural synthesis and biosynthetic mechanisms of these compounds. Increasing research indicates that organisms may naturally biosynthesize spontaneous conjugates with structures similar to DPCs. A prime example is N‐retinylidene‐phosphatidylethanolamine (N‐Ret‐PE).^152^ N‐Ret‐PE is formed through a reversible Schiff base addition reaction between all‐trans retinal and phosphatidylethanolamine (PE), playing a critical role in the visual cycle. Gaining an understanding of such DPC biosynthesis is crucial for elucidating various physiological processes.^153^

Although DPCs have been widely studied and shown good safety and efficacy both in vitro and in vivo, very few have achieved clinical translation and FDA approval. This may be due to the relatively short research period for most DPCs and the need for further validation of their in vivo metabolism. However, some DPCs have been rapidly approved by the FDA and translated clinically due to their significantly enhanced efficacy. A typical example is DP‐VPA, used for treating epilepsy, which is considered a safer and more effective alternative to valproic acid.^142^, ^143^ Additionally, CLR131, a conjugate of radioactive iodine and phospholipids, was also quickly approved by the FDA for the treatment of hematologic cancers.^154^ With continued research, we have reason to believe that more DPCs will achieve breakthrough progress in clinical translation.

With the advancement of synthesis technologies, the molecular design of DPCs is becoming increasingly diverse, and their potential in the field of drug delivery is growing daily. Future research will further explore the efficacy and safety of DPCs in clinical applications as well as how to optimize their structures to accommodate a wider variety of disease types and treatment requirements.

## AUTHOR CONTRIBUTIONS

Yawei Du and Wenguo Cui conceived the idea; Ding Zhao and Tianqi Wu wrote the manuscript; Yixiang Zhang, Fan Wang and Rames Kaewmanee revised the manuscript.

## CONFLICT OF INTEREST STATEMENT

The authors declare that there are no competing interests.
